# Reply to Dhawan et al. Comment on “Kim et al. The Association between Coffee Consumption and Risk of Colorectal Cancer in a Korean Population. *Nutrients* 2021, *13*, 2753”

**DOI:** 10.3390/nu14010030

**Published:** 2021-12-23

**Authors:** Youngyo Kim, Jeongseon Kim

**Affiliations:** 1 Department of Food and Nutrition/Institute of Agriculture and Life Science, Gyeongsang National University, Jinju 52828, Korea; youngyokim@gnu.ac.kr; 2 Department of Cancer Biomedical Science, Graduate School of Cancer Science and Policy, National Cancer Center, Goyang 10408, Korea

We would like to thank you for the interest in our article [1]. We agree with the comments on the limitations of the studies [2]. 

We considered coffee additives to be a confounding factor, as the real association between coffee consumption and colorectal cancer could be influenced by Koreans’ high intake of instant coffee mixes containing sugar and cream additives. At first, we thought that it would be best to analyze the sample by separating the people based on whether they supplemented coffee with sugar and cream as the previous study targeted for the Korean population [3]. However, we could not analyze by coffee type (i.e., filtered coffee without cream and sugar/instant coffee including cream and sugar) because our food frequency questionnaire did not investigate by coffee type. Instead, we chose to analyze by adjusting for the amount of cream and sugar added to coffee referring to the previous study by Gardener et al. [4] and evaluated the multicollinearity by checking the variance inflation factor and the condition index.

We agree that comparing coffee drinkers with high additive intake and separating coffee drinkers with low additive intake with non-drinkers of coffee will be important for future work in order to avoid potential collinearity.
